# Associations between periodontal inflammation and OHIP‐14 scores: A cross‐sectional study

**DOI:** 10.1002/jper.70165

**Published:** 2026-08-06

**Authors:** Luigi Nibali, Yanwen Zheng, Koula Asimakopoulou, Jeremy Redman, Alastair Lomax, Jing Kang

**Affiliations:** ^1^ Periodontology Unit, Centre for Host Microbiome Interactions, Faculty of Dentistry, Oral & Craniofacial Sciences King's College London London UK; ^2^ Centre of Oral, Clinical and Translational Sciences, Faculty of Dentistry, Oral & Craniofacial Sciences King's College London London UK; ^3^ Faculty of Health Science and Technology Oxford Brookes University Oxford UK; ^4^ Lewisham London UK; ^5^ Oral Healthcare HALEON Weybridge UK

**Keywords:** inflammation, periodontitis, quality of life

## Abstract

**Background:**

More evidence is needed to assess the association between periodontitis and Oral Health‐Related Quality of Life (OHRQoL) quantitatively. We hypothesized that periodontal inflammation is correlated with a worsening in quality of life and aimed to test this in this study.

**Methods:**

A total of 1307 participants in King's Oral Biobank answered the Oral Health Impact Profile‐14 (OHIP‐14) questionnaire (measurement of OHRQoL) during a dental visit. The associations between periodontal inflammation, measured as periodontal inflamed surface area (PISA) and bleeding on probing (BOP), periodontal conditions (diagnosis/staging), and OHIP‐14 scores across all seven domains (functional limitation, physical pain, psychological discomfort, physical disability, psychological disability, social disability, and handicap) were investigated.

**Results:**

The PISA exhibited weak linear correlations with the overall OHIP‐14 score (Pearson correlation coefficient *r* = 0.06, *p* = 0.037), particularly with the “physical pain” subdomain (*r* = 0.12, *p* < 0.001). BOP was associated with the OHIP overall score (*r* = 0.08, *p* = 0.022). In addition, stage IV periodontitis was associated with higher OHIP‐14 total scores and with multiple subdomains, including physical pain, physical disability and psychological discomfort.

**Conclusions:**

Inflammation indices such as BOP and PISA are associated with worse OHRQoL. In addition, the complex clinical features of stage IV periodontitis may worsen OHRQoL, especially in relation to physical pain, physical disability and psychological discomfort.

**Plain language summary:**

This study shows that having more advanced gum disease (stage IV) and gum inflammation may worsen quality of life.

## 1 INTRODUCTION

Quality of life is defined by the World Health Organization as “individuals’ perception of their position in life in the context of the culture and value systems in which they live and in relation to their goals, expectations, standards and concerns”. The concept of oral health‐related quality of life (OHRQoL) is a measure of quality of life related to oral health, and it provides a subjective assessment of an individual's view of their oral health, taking into accounts their perceptions of biological, social, psychological, and cultural characteristics.^1^

Periodontitis is characterized by gingival inflammation and tissue destruction, resulting in halitosis, gingival swelling and/or recession, tooth sensitivity, mobility, drifting of teeth and, eventually, tooth loss. As such, periodontitis has been shown to affect patients’ OHRQoL across several domains, ranging from functional to psycho‐social aspects.^2^, ^3^, ^4^ The Oral Health Impact Profile‐14 (OHIP‐14) questionnaire is a relatively simple system for assessing the impacts of oral diseases on the daily life of people over a predetermined period, including 14 questions spanning seven domains: functional limitation, physical pain, psychological discomfort, physical disability, psychological disability, social disability and handicap.^5^ Each participant is asked to indicate whether each impact has affected them at one of five levels: “never”, “hardly ever”, “occasionally”, “fairly often”, or “very often”.

Despite several systematic reviews assessing associations between periodontitis and OHIP‐14 scores,^2^, ^3^, ^4^, ^6^ it is not clear how much of the perceived effects of periodontal disease are particularly due to the level of inflammation in each individual case, or to other related issues such as mobility, masticatory dysfunction, or pain. Measures of periodontal inflammation include clinical assessment using bleeding on probing, gingival index, or biomarkers such as levels of inflammatory cytokines in gingival crevicular fluid or saliva. Another measure of periodontal inflammation, which incorporates bleeding and the amount of tissue destruction is the periodontal inflamed surface area (PISA).^7^ So far, to our knowledge, no published study has investigated the associations between PISA and OHRQoL in a large cohort of patients with different periodontal phenotypes and demographic backgrounds.

Therefore, the aim of this study is to investigate the association between periodontal inflammation (measured by PISA score and bleeding on probing, BOP), disease severity (periodontal diagnosis, and periodontal staging) and oral health‐related quality of life, using the clinical data collected in our in‐house Biobank.

## 2 MATERIALS AND METHODS

The primary objective of this cross‐sectional study was to test the association between periodontal inflammation as measured by PISA^7^ and OHRQoL (measured with OHIP‐14). The key secondary objectives were to test the association between OHIP‐14 scores and BOP, and periodontal diagnosis/staging.

The Strengthening the Reporting of Observational Studies in Epidemiology (STROBE) guidelines, available in the Supporting Information section (Supplemental material 1), were followed. This investigation was carried out with support from a patient representative (author Jeremy Redman), who assisted the study from planning to manuscript preparation.

### 2.1 Patient population and ethical approval

Samples were collected from patients taking part in the King's College London Oral, Dental and Craniofacial Biobank. The Biobank was granted ethics approval by the East of England‐Cambridge East Research Ethics Committee (reference 20/EE/0241). The study was conducted according to the principles of the Declaration of Helsinki. Each patient gave written consent to take part in the Biobank recruitment in the Periodontology new patients’ clinic at the Guy's & St Thomas Foundation Trust Hospital (GSTFT). Specific approval for the release of data and samples for this study was granted by the Biobank Management Committee (Biobank reference 026). The study sample size was a convenient sample based on available Biobank data.

#### 2.1.1 Patients’ inclusion criteria

− Age ≥ 18 years old;
− Participants who gave written consent to be included in King's College London Oral, Dental and Craniofacial Biobank from October 2020 to November 2024; and
− Participants who filled in the OHIP‐14 questionnaire and had measurements of periodontal disease collected.

### 2.2 Exposure

The main exposure in this analysis is the PISA score, reflecting periodontal inflammation derived from clinical examination. Other exposures include BOP, periodontal diagnosis and periodontal staging, as described below.

The following clinical periodontal measurements were taken at six tooth sites by Biobank examiners using a UNC‐15 periodontal probe: dichotomous full mouth plaque scores (FMPS); full mouth probing pocket depth (PPD); recession (REC) of the gingival margin from the cemento‐enamel junction (CEJ); BOP^8^; tooth mobility^9^; and furcation involvement.^10^ Clinical attachment level (CAL) was calculated as PPD+REC.

#### 2.2.1 PISA score

PISA score was calculated as previously described using the clinical measures including PPD, BOP, REC, and CAL described above.^7^

#### 2.2.2 Periodontal diagnosis

Periodontal diagnosis was based on the current classification of periodontal diseases,^11^ using the following criteria:

- Periodontal health: BOP < 10% and PPD ≤4 mm and no site ≥4 mm PPD with BOP with evidence of previous bone loss/attachment loss.
- Gingivitis: BOP ≥10% with all sites with PPD ≤3 mm.
- Periodontitis: presence of ≥2 non‐adjacent sites with PPD = 4 mm and BOP or > 4 mm PPD with evidence of radiographic bone loss (except in the case of deep caries, endo‐periodontal pathology, fracture, third molars). Patients with periodontitis were further subdivided according to staging and grading^11^.

### 2.3 Outcome: OHRQoL

OHRQoL was measured by OHIP‐14. This a reliable, valid measure^12^ used to assess overall quality of life related to oral health but also to provide information on seven subdomains: (1) Functional Limitation (how oral health problems affect a person's ability to perform daily tasks like eating or speaking); (2) Physical Pain (assessing the experience of pain that is attributed to oral health issues); (3) Psychological Discomfort (assessing the patient's levels of worry or distress related to oral health issues); (4) Physical Disability (assessing how oral health problems limit the person's physical activities); (5) Psychological Disability (assessing the extent to which oral health issues affect a person's psychological state, such as feeling self‐conscious); (6) Social Disability (looking at how oral health problems might affect the person's impact social interactions with others); and finally (7) Handicap (where the measure evaluates the overall impact of oral health problems on a person's ability to participate in broad social roles and activities).

The OHRQoL outcome as measured by OHIP‐14 score can be presented in four formats: (1) total OHIP‐14 score [the sum of all scores from 0 (never) to 4 (very often), scale range 0–28]; (2) each of the seven OHIP domain described above, with each domain scale range 0–8; (3) FOVO prevalence (presence of at least one response of “Fairly Often” or “Very Often” to any of the 14 OHIP questions); (4) OFOVO prevalence (presence of at least one response of “Occasionally,” “Fairly Often” or “Very Often” to any of the 14 OHIP questions).^13^

The OHIP‐14 questionnaire was given to each Biobank participant at the study visit prior to the clinical assessment. The questionnaires were then collected and data were entered into an Excel spreadsheet for further data analyses and proof‐read for entry errors.

### 2.4 Covariates

Following patients’ consent, demographic (socio‐economic status, sex, age, ethnicity) and medical particulars were collected, along with dental and medical history. Self‐reported smoking habits were recorded (number of cigarettes per day, years of smoking). Height, weight, and waist measurements were taken at the study visit and body mass index (BMI) derived from those measurements. All included covariates were evidenced to be associated with periodontal disease and OHRQoL.^6^

### 2.5 Statistical analysis

Descriptive statistics were performed for each of the collected variables including participants’ demographic background, medical condition, lifestyle, periodontal inflammatory condition and oral health related quality of life. Scatter plots with calculated Pearson correlations coefficient *r* illustrating the association between the PISA score and OHIP were performed, as well as their association in each OHRQoL domain. Pearson correlation coefficient *r* of 0.90–1.00 indicates very strong, 0.70–0.89 as strong, 0.40–0.69 as moderate, 0.1–0.39 as weak, and 0.00–0.10 as negligible associations.

Linear regression models were used to evaluate the association between periodontal inflammation (PISA score) and OHIP‐14 outcome, using univariable and multi‐variable approaches with adjustment of covariates (age, sex, ethnicity, smoking status, presence of systemic conditions, number of teeth). Additional regression models were also performed using periodontal diagnosis (healthy, gingivitis, periodontitis) or periodontal staging (stages 0–IV) as predictor to assess their association with OHIP‐14 outcome. Missing data were handled under the Missing at Random (MAR) assumption using multivariate imputation by chained equations (MICE). Five imputed datasets were generated, and pooled estimates were calculated using Rubin's rules to obtain unbiased parameter estimates and standard errors.^14^, ^15^ Multicollinearity among covariates was assessed using variance inflation factors (VIFs). A VIF value below 5 indicates a low multicollinearity issue. In addition, it is important to interpret the clinical significance and not just statistical significance of patient‐reported outcome measures (PROMs), for example, by using the “minimally important difference” (MID).^13^

OHRQoL outcomes measured by other formats such as OFOVO, FOVO, and by each domain, were also examined in relation to their association with periodontal inflammation (PISA score and BoP) or periodontal phenotype (health, localized gingivitis, generalized gingivitis, periodontitis from stage I to IV).

All statistical analyses were conducted using R (version 4.3.2; R Foundation for Statistical Computing) via RStudio for Windows (version 2025.05.1‐513). All regression estimates were derived from pooled imputed datasets, and statistical significance was set at 0.05.

## 3 RESULTS

### 3.1 Participant characteristics

A total of 1307 participants [mean age 51.80 years, standard deviation (SD) 14.50] were included in the analysis, after excluding those with missing vital clinical data and those without consent or under 18 years old. The analysis flow chart was presented in Supplemental material 2, and the basic demographic and clinical information about the participants were presented in Table 1. Participants’ characteristics by periodontal diagnosis condition were further examined and presented as Supplemental material 3.

**TABLE 1:** Baseline characteristics of participants (*N* = 1,307).

Age (years) (mean (SD))	51.80 (14.50)
Sex = Female	739 (56.54%)
Ethnicity	
Caucasian	742 (56.77%)
Afro‐Caribbean	272 (20.81%)
Asian	196 (15.00%)
Other‐mixed	71 (5.43%)
Smoking status	
Never	705 (53.94%)
Former	383 (29.30%)
Current	211 (16.14%)
BMI (kg/m^2^) (mean (SD))	26.75 (6.87)
Any systemic condition = Yes	727 (55.62%)
Diabetes = Yes	142 (10.86%)
Cardiovascular disease = Yes	169 (12.93%)
Hypertension = Yes	275 (21.04%)
Rheumatoid arthritis = Yes	85 (6.50%)
No. of teeth (mean (SD))	26.11 (5.26)
Periodontal stage	
Stage 0 (healthy)	199 (15.23%)
Stage I	25 (1.91%)
Stage II	92 (7.04%)
Stage III	561 (42.92%)
Stage IV	353 (27.01%)
OHIP total score (mean (SD))	13.84 (11.41)
OHIP‐14: OFOVO prevalence	4.54 (3.89)
OHIP‐14: FOVO prevalence	1.64 (2.61)
PISA score (mm^2^) (mean (SD))	418.87 (650.90)

*Note*: Values are presented as number (percentage) unless stated otherwise, based on available (non‐missing) data. Group comparisons were performed using one‐way ANOVA or Kruskal–Wallis tests for continuous variables and chi‐square tests or Fisher's Exact tests for categorical variables, as appropriate. There were 58 (4.4%) participants with no information of the periodontal diagnosis hence omitted in the table. Other missing data included: BMI (7.3%), periodontal stage (5.9%), number of teeth (3.3%), ethnicity (2%), systemic and related conditions (≤1.1%), PISA score (0.8%), smoking (0.6%), and sex (0.3%).

Abbreviations: ANOVA, analysis of variance; BMI, body mass index; CVD, cardiovascular disease; FOVO, items rated “fairly often” or “very often” (score ≥ 3); OFOVO, items rated “occasionally”, “fairly often”, or “very often” (score ≥ 2); OHIP, Oral Health Impact Profile; PD, periodontal disease; PISA, periodontal inflamed surface area.

### 3.2 Periodontal inflammation and OHIP‐14

The association between PISA score and OHIP‐14 total scores is shown in the Figure 1 scatter plot, with Pearson correlation coefficient = 0.059, 95% confidence interval, CI 0.004–0.114, *p* = 0.037. This result, while statistically significant, reveals a negligible magnitude of correlation. Figure 2 further demonstrates the association between PISA score and each domain of OHIP‐14. It is noted that the physical pain domain showed the highest correlation with PISA score, albeit with a very weak correlation. When examining the association between PISA and OHIP, these two were weakly correlated in periodontitis group (*r* = 0.06, *p* = 0.04), but this association was not observed in gingivitis group or health group (Supplemental material 4).

**FIGURE 1:**
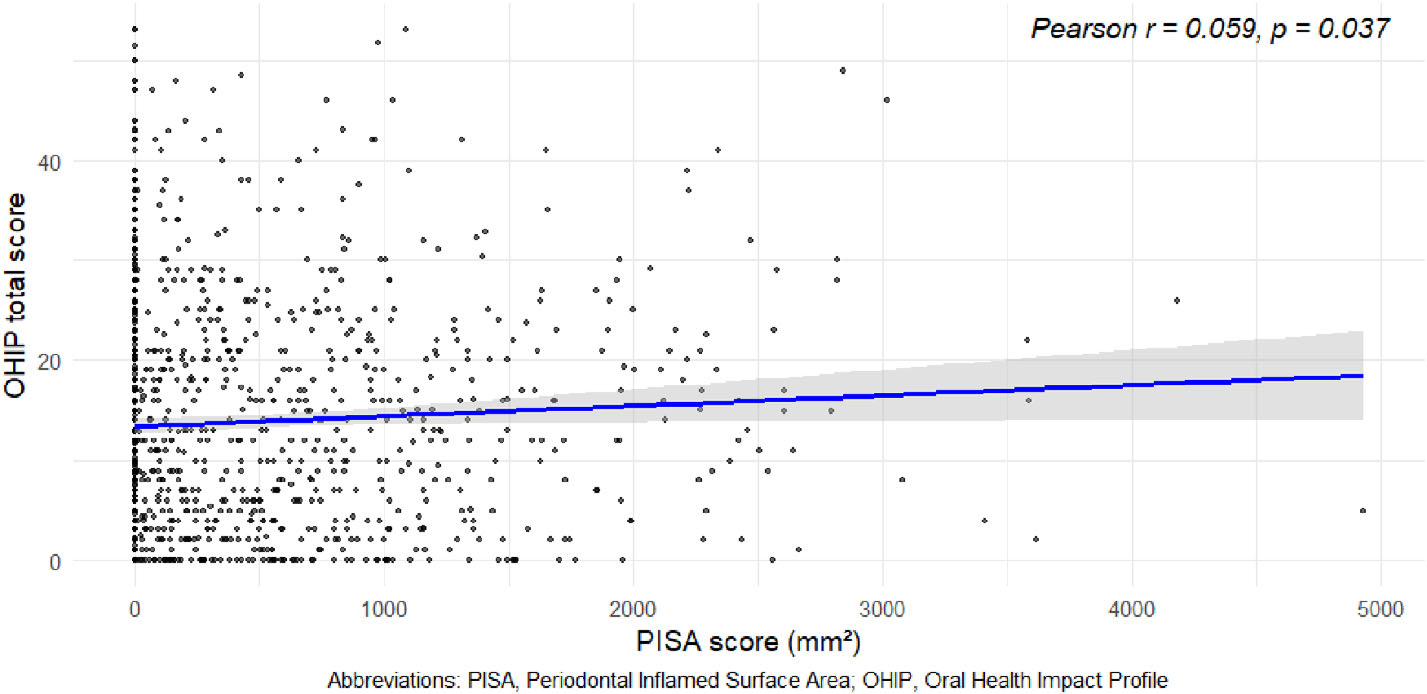
Scatterplot showing the association between PISA score and OHIP total score, with fitted line and correlation coefficients (Pearson). OHIP, Oral Health Impact Profile; PISA, periodontal inflamed surface area.

**FIGURE 2:**
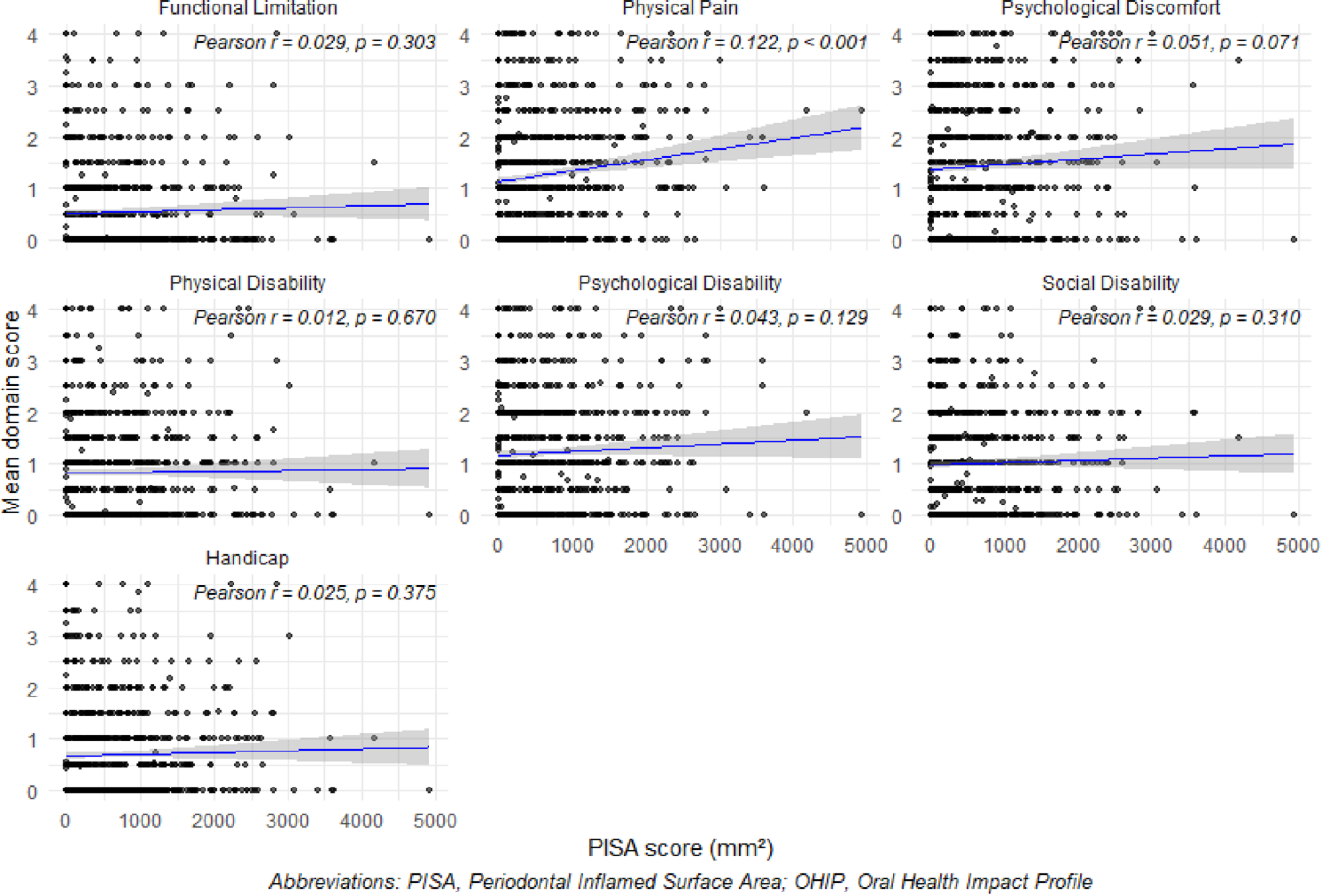
Scatterplot showing the association between PISA score and each OHIP domain, with regression line and correlation coefficients (Pearson). OHIP, Oral Health Impact Profile; PISA, periodontal inflamed surface area.

BOP was weakly associated with OHIP overall score in the whole cohort (*r* = 0.08, *p* = 0.022), but not in diagnosis subgroups (healthy, gingivitis, periodontitis) (Supplemental material 5).

### 3.3 Oral impact by periodontitis diagnosis and staging

The number of OHIP‐14 items rated as FOVO was higher in the periodontitis group (mean = 1.61 ± 2.59) than the gingivitis (1.20 ± 2.02) and healthy (1.32 ± 2.59) groups (*p* = 0.042) (Figure 3). Furthermore, item‐level analysis showed a progressive increase in FOVO response rates for Q5 (“feeling self‐conscious”) and Q11 (“life less satisfying”) across the periodontal diagnosis groups (Figure 3).

**FIGURE 3:**
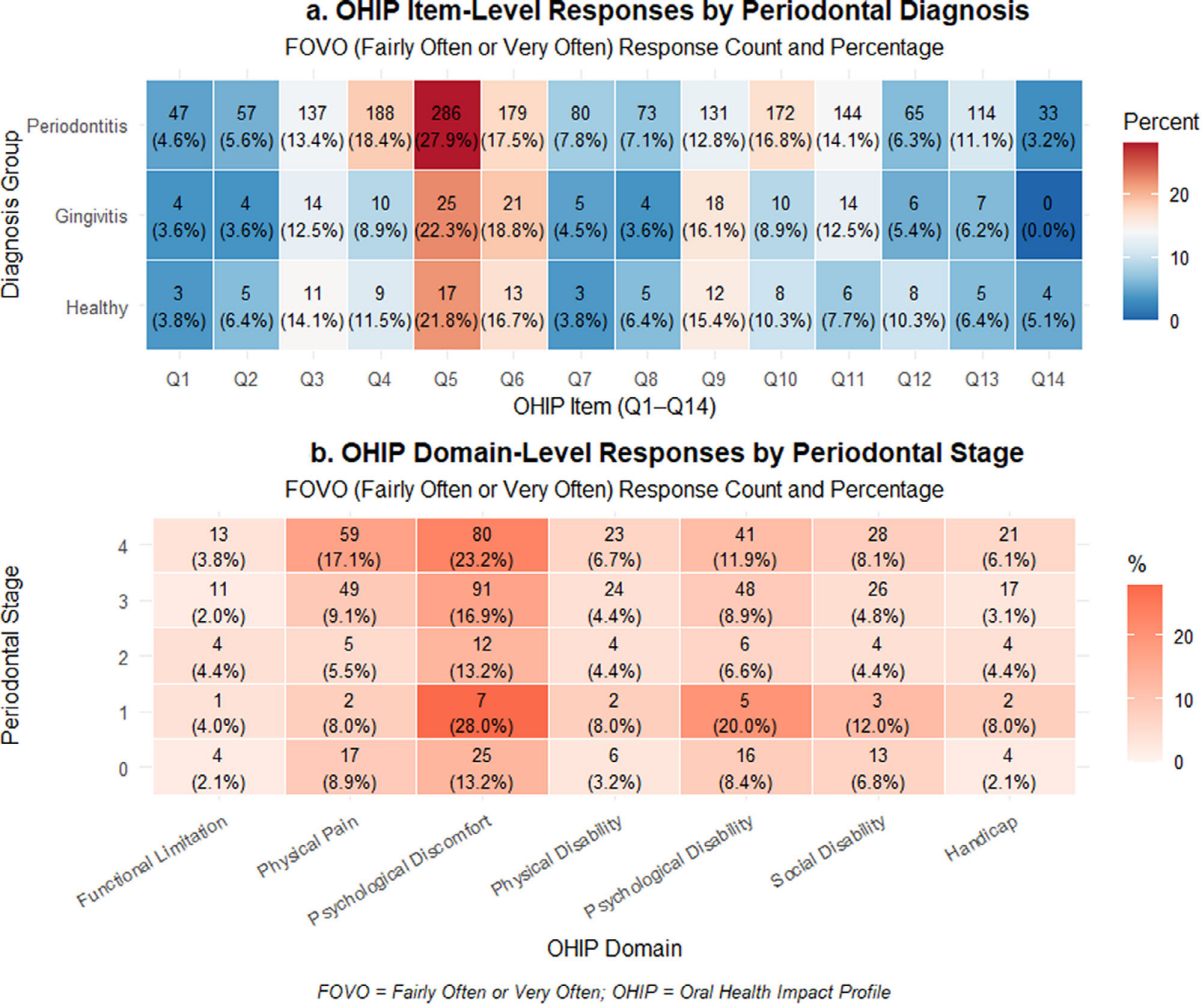
OHIP “fairly often or very often” item‐level responses by periodontal diagnosis group (A) and subdomain‐level responses by periodontal stage (B). Figure 3A displays item‐level OHIP‐14 responses rated as “fairly often” or “very often” (FOVO, score ≥ 3) across periodontal diagnosis groups (healthy, gingivitis, periodontitis). Figure 3B presents aggregated domain‐level FOVO responses by periodontal stage (0–IV). Each cell in the heatmaps shows both the count and percentage of participants with FOVO responses. FOVO, fairly often or very often; OHIP‐14, Oral Health Impact Profile‐14; OHRQoL, oral health‐related quality of life.

Correlations between PISA and OHIP domains across different periodontal diagnosis groups are summarized in Supplemental material 6.

### 3.4 Effect of PISA on OHRQoL using linear regression models

Among all participants, the adjusted effect of PISA on OHIP‐14 is 0.03 (95% CI −0.07 to 0.13). Physical pain was positively associated with PISA (effect size = 0.03, 95% CI 0.009–0.048 after adjusting diagnosis group), but this association was attenuated after further adjustment by periodontal stages. Among participants with periodontitis, an association between PISA and OHIP was observed (*r* = 0.11, 95%, CI 0.005–0.209), but not after adjusting for periodontal staging. Physical pain domain in OHIP remains the key area where PISA and OHIP are associated (Table 2).

**TABLE 2:** The effect of PISA score on OHIP‐14 total score, and by each domain.

Parameter	OHIP‐14 total	Functional limitation	Physical pain	Psychological discomfort	Physical disability	Psychological disability	Social disability	Handicap
**All participants (*N* = 1,307)**								
PISA per 100mm^2^, crude	0.096	0.008	0.038	0.018	0.003	0.015	0.01	0.005
	(−0.004, 0.195)	(−0.007, 0.022)	(0.019, 0.058)^***^	(−0.005, 0.040)	(−0.014, 0.019)	(−0.005, 0.034)	(−0.007, 0.028)	(−0.011, 0.020)
PISA per 100mm^2^, adjusted^0^	0.074	0.009	0.031	0.014	0	0.01	0.007	0.003
	(−0.022, 0.170)	(−0.006, 0.024)	(0.012, 0.050)^**^	(−0.008, 0.035)	(−0.018, 0.018)	(−0.009, 0.029)	(−0.011, 0.024)	(−0.013, 0.020)
PISA per 100mm^2^, adjusted^1^	0.068	0.01	0.029	0.012	−0.002	0.01	0.008	0.002
	(−0.030, 0.166)	(−0.005, 0.026)	(0.009, 0.048)^**^	(−0.010, 0.034)	(−0.020, 0.016)	(−0.010, 0.029)	(−0.010, 0.026)	(−0.015, 0.019)
PISA per 100mm^2^, adjusted^2^	0.03	0.007	0.018	0.004	−0.006	0.005	0.003	−0.001
	(−0.070, 0.130)	(−0.008, 0.023)	(−0.002, 0.038)	(−0.019, 0.026)	(−0.024, 0.013)	(−0.015, 0.025)	(−0.015, 0.021)	(−0.019, 0.016)
**Participants with periodontitis only (*n* = 1,054)**								
PISA per 100mm^2^, crude	0.107	0.009	0.043	0.019	0.005	0.016	0.01	0.004
	(0.005, 0.209)^*^	(−0.005, 0.024)	(0.022, 0.063)^***^	(−0.004, 0.042)	(−0.012, 0.023)	(−0.004, 0.036)	(−0.007, 0.028)	(−0.012, 0.021)
PISA per 100 mm^2^, adjusted^0^	0.064	0.011	0.031	0.011	−0.002	0.008	0.007	−0.001
	(−0.036, 0.165)	(−0.005, 0.026)	(0.011, 0.051)^**^	(−0.012, 0.034)	(−0.021, 0.016)	(−0.012, 0.028)	(−0.011, 0.025)	(−0.019, 0.017)

*Note*: N = 1,307. Missing data imputed using multiple imputation techniques.

Abbreviations: OHIP‐14, Oral Health Impact Profile‐14; PISA, periodontal inflamed surface area.

^0^ Covariates adjusted in the linear regression model 0: age, sex, ethnicity, smoking status, number of teeth, and systemic comorbidities (cardiovascular disease, hypertension, diabetes, rheumatoid arthritis).

^1^ Further adjusted by diagnosis group in addition to covariate in model 0.

^2^ Further adjusted by periodontal stages in addition to covariates used in model 0.

Asterisks indicate statistical significance: *p* < 0.05 (^*^), *p* < 0.01 (^**^), *p* < 0.001 (^***^).

### 3.5 Effect of periodontal diagnosis and periodontal stages on OHRQoL using linear regression models

The effect size of periodontal diagnosis (gingivitis vs. healthy, periodontitis versus healthy, where healthy group is the reference) of OHIP outcome is presented in Supplemental material 7. Overall OHIP‐14 score and each OHIP domain are presented, using univariable and multiple variable approaches adjusting the covariates mentioned before. Compared to the healthy group, the periodontitis group and gingivitis group did not show a different OHIP score.

A similar analysis was performed for periodontal stages (0 to IV), where stage 0 is used as reference group. Periodontal stage IV showed a significantly higher OHIP total score and in the sub‐domains of physical pain, psychological discomfort, and physical disability (Supplemental material 8) in the adjusted models.

All VIFs were found to be below 5, suggesting that multicollinearity was not a concern in the multivariable regression models.

## 4 DISCUSSION

This study presents evidence on how gingival inflammation may directly contribute to worse OHRQoL in gingivitis and periodontitis patients. In addition, it suggests that periodontal disease severity is associated with worse OHRQoL, as measured by the OHIP‐14. This association, related to stage IV periodontitis, which seems to impact these patient‐reported outcomes, suggests that the factors shifting from stage III to IV have an important impact in worsening oral health‐related quality of life. However, it is important to notice that, despite statistical significance, the magnitude of the observed linear associations was weak, meaning that other factors probably have a stronger impact on OHIP‐14 scores.

The data reported herein contribute to the existing evidence from PROMS and periodontal disease and largely confirm previous findings, albeit with a larger sample size than most previous studies. In particular, the reverse association between periodontitis and OHRQoL was reported in a systematic review a decade ago^2^ and confirmed by several studies since.^4^, ^6^ It has also been reported that these quality‐of‐life measures appear to improve following periodontal treatment.^16^ It is important to acknowledge that the main analysis of all OHIP‐14 data across periodontal phenotypes (health–gingivitis–periodontitis) in the present study did not reach a statistically significant level, while periodontitis was significantly associated only with the “physical pain” subdomain (*β* = 0.40, *p* < 0.05). This correlates with a recent systematic review, showing that physical and psychological dimensions are the most affected in periodontitis.^4^

The present study aimed to investigate in more detail the effect of periodontal inflammation on overall and different domains of OHRQoL. The linear association between PISA score and OHIP‐14 total score was statistically significant, albeit weak, in the entire sample. While this could be affected by PPDs, attachment loss and downstream consequences (mobility, discomfort), in cases of gingivitis, inflammation is the hallmark and probably the main contributor to the quality‐of‐life measures. This suggests that gingival inflammation, especially when more generalized, may lead to discomfort and disability. In fact, it has been suggested that periodontitis may exert a greater effect on OHRQoL in the presence of gingival bleeding, halitosis and tooth mobility.^17^ However, it is difficult to compare this with existing studies, as very little information exists on the effects of gingivitis on OHRQoL measures, particularly in adults.^18^ In a study on adolescents in Brazil, gingivitis at baseline was associated with worse overall OHRQoL measures and worse emotional well‐being, independently of other oral conditions and socioeconomic variables.^19^ Similar results were found in a study on 12‐year‐old Ecuadorian schoolchildren, for which gingival bleeding negatively impacted the OHRQoL.^20^ A study including 323 participants in Turkey found that patients with periodontitis had poorer OHRQoL than gingivitis patients. However, no healthy controls were included.^21^ More specific OHRQoL questions, which encompass symptoms of inflammation such as bleeding gums, may generate clearer data on the effects of gingivitis.

The other important element worth of discussion is related to stage IV periodontitis, which was significantly associated with higher OHIP‐14 total scores and with multiple subdomains, including physical pain, physical disability and psychological discomfort. This is not surprising and supports previous smaller studies, such as one including 166 patients, showing that patients with stage IV and grade C periodontitis had the highest total OHIP‐14 scores and symptoms of bleeding gums, sore gums, swollen gums, bad breath, loose teeth and drifting teeth.^22^ Similarly, a cross‐sectional study of 214 patients in Hong Kong showed that patients with stage IV periodontitis reported reduced food intake or altered food type intake attributed to difficulties in chewing, objective measures of masticatory dysfunction, tooth loss, as well as more advanced periodontal breakdown compared with subjects with stages I‐III.^23^ A recent systematic review found worse OHIP‐14 scores in individuals with stages III–IV periodontitis compared with stages I–II periodontitis, although in many cases diagnoses had been assigned retrospectively.^4^ In another systematic review, pooled results suggested a trend toward higher differences in OHIP‐14 total scores between the periodontitis groups in advanced stages.^6^

These findings are in line with the concept that periodontitis patients experience challenges in basic daily activities, such as eating and speaking, and lend weight to the importance of the factors contributing to the shift from stage III to stage IV periodontitis. The complexity factors which contribute to the definition of stage IV periodontitis, such as previous tooth loss, reduced biting pairs, presence of hypermobile teeth, and tooth drifting are clearly reflected in patient symptoms such as physical pain, physical disability and psychological discomfort. Among other items, the responses to Q5 (feeling self‐conscious) and Q11(“life less satisfying”) exhibited significant step increases across the three groups, again suggesting the influence of periodontal disease on these aspects. However, these associations appeared rather weak, suggesting that other factors have stronger impacts on oral health related QoL.

Among other factors, female sex, smoking and the presence of systemic diseases were also independently associated with worse OHRQoL, which also mirrors results from previous studies.^6^

The main strength of this study is its relatively large sample size compared with most previous studies. It also benefits from input by a patient representative, adding an important dimension to the interpretation of results. However, the limitations are the use of a very basic OHRQoL questionnaire, which may not really capture more nuanced details related to patient symptoms and the fact that not all healthy and gingivitis cases were free of other dental diseases, which may also affect quality of life. The structure of the OHIP‐14 remains a subject of debate. However, it is recognized as a convenient and reliable short‐scale instrument.^6^

## 5 CONCLUSION

Overall, the present study offers evidence to support the notion that the clinical features of stage IV periodontitis are associated with PROMS related to physical pain, physical disability and psychological discomfort. However, simple measures of gingival inflammation such as BOP or PISA are also weakly associated with worse OHRQoL. The importance of PROMS and particularly OHRQoL is now consistently recognized and an increasing number of studies have implemented it as an important outcome. In clinical settings, it may be worth to routinely assess OHRQoL in patients with periodontal disease and to reduce gingival inflammation and downstream consequences such as tooth mobility, drifting and tooth loss (typical of stage IV periodontitis) to potentially reduce impacts on patients’ quality of life. Our research group has recently proposed combining PROMS outcomes with clinical measures, better to assess responses to treatment.^24^

Further studies should investigate more specifically the associations between clinical signs of disease and patient symptoms, particularly in the understudied field of gingivitis in adults.

## AUTHOR CONTRIBUTIONS

**Yanwen Zheng**: Investigation; writing—review and editing; methodology; formal analysis; data curation. **Alastair Lomax**: Conceptualization; investigation; funding acquisition; writing—review and editing. **Jeremy Redman**: Investigation; writing—review and editing. **Luigi Nibali**: Conceptualization; methodology; investigation; funding acquisition; writing—original draft; writing—review and editing; project administration; supervision. **Jing Kang**: Investigation, formal analysis; supervision; methodology; writing—review and editing. **Koula Asimakopoulou**: Writing—review and editing; methodology; conceptualization; funding acquisition.

## CONFLICT OF INTEREST STATEMENT

The authors declare no conflicts of interest.

## CONSENT STATEMENT

Each patient gave written consent prior to inclusion in the study.

## Supporting information




**Supporting information**: jper70165‐sup‐0001‐SupMat.docx
