# Supplementary material for: Associations between periodontal inflammation and OHIP‐14 scores: A cross‐sectional study
Source: J Periodontol. 2026 Aug 6;97(10):2150–9. doi: 10.1002/jper.70165 (PMC13743496; doi:10.1002/jper.70165)
Supplement: Supplementary file 1 [file JPER-97-2150-s001.docx]

**Supplemental materials**

**Supplemental material 1**

STROBE Statement—Checklist of items that should be included in reports of ***cross-sectional studies***

|  | Item No | Recommendation | Page No |
| --- | --- | --- | --- |
| **Title and abstract** | 1 | (*a*) Indicate the study’s design with a commonly used term in the title or the abstract | 1 |
|  |  | (*b*) Provide in the abstract an informative and balanced summary of what was done and what was found | 2 |
| Introduction | | | |
| Background/rationale | 2 | Explain the scientific background and rationale for the investigation being reported | 3 |
| Objectives | 3 | State specific objectives, including any prespecified hypotheses | 3 |
| Methods | | | |
| Study design | 4 | Present key elements of study design early in the paper | 4 |
| Setting | 5 | Describe the setting, locations, and relevant dates, including periods of recruitment, exposure, follow-up, and data collection | 4 |
| Participants | 6 | (*a*) Give the eligibility criteria, and the sources and methods of selection of participants | 4 |
| Variables | 7 | Clearly define all outcomes, exposures, predictors, potential confounders, and effect modifiers. Give diagnostic criteria, if applicable | 4-5 |
| Data sources/ measurement | 8* | For each variable of interest, give sources of data and details of methods of assessment (measurement). Describe comparability of assessment methods if there is more than one group | *5* |
| Bias | 9 | Describe any efforts to address potential sources of bias | 5-6 |
| Study size | 10 | Explain how the study size was arrived at | 5 |
| Quantitative variables | 11 | Explain how quantitative variables were handled in the analyses. If applicable, describe which groupings were chosen and why | 6 |
| Statistical methods | 12 | (*a*) Describe all statistical methods, including those used to control for confounding | 6 |
|  |  | (*b*) Describe any methods used to examine subgroups and interactions | 6 |
|  |  | (*c*) Explain how missing data were addressed | 6 |
|  |  | (*d*) If applicable, describe analytical methods taking account of sampling strategy | n/a |
|  |  | (*e*) Describe any sensitivity analyses | 6 |
| Results | | | |
| Participants | 13* | (a) Report numbers of individuals at each stage of study—eg numbers potentially eligible, examined for eligibility, confirmed eligible, included in the study, completing follow-up, and analysed | 6 |
|  |  | (b) Give reasons for non-participation at each stage | 6 |
|  |  | (c) Consider use of a flow diagram | 6 |
| Descriptive data | 14* | (a) Give characteristics of study participants (eg demographic, clinical, social) and information on exposures and potential confounders | 7 |
|  |  | (b) Indicate number of participants with missing data for each variable of interest | 7 |
| Outcome data | 15* | Report numbers of outcome events or summary measures | 7 |
| Main results | 16 | (*a*) Give unadjusted estimates and, if applicable, confounder-adjusted estimates and their precision (eg, 95% confidence interval). Make clear which confounders were adjusted for and why they were included | 7-8 |
|  |  | (*b*) Report category boundaries when continuous variables were categorized | n/a |
|  |  | (*c*) If relevant, consider translating estimates of relative risk into absolute risk for a meaningful time period | n/a |
| Other analyses | 17 | Report other analyses done—eg analyses of subgroups and interactions, and sensitivity analyses | 7 |
| Discussion | | | |
| Key results | 18 | Summarise key results with reference to study objectives | 8 |
| Limitations | 19 | Discuss limitations of the study, taking into account sources of potential bias or imprecision. Discuss both direction and magnitude of any potential bias | 9 |
| Interpretation | 20 | Give a cautious overall interpretation of results considering objectives, limitations, multiplicity of analyses, results from similar studies, and other relevant evidence | 10 |
| Generalisability | 21 | Discuss the generalisability (external validity) of the study results | 10 |
| Other information | | | |
| Funding | 22 | Give the source of funding and the role of the funders for the present study and, if applicable, for the original study on which the present article is based | 1 |

*Give information separately for exposed and unexposed groups.

**Note:** An Explanation and Elaboration article discusses each checklist item and gives methodological background and published examples of transparent reporting. The STROBE checklist is best used in conjunction with this article (freely available on the Web sites of PLoS Medicine at http://www.plosmedicine.org/, Annals of Internal Medicine at http://www.annals.org/, and Epidemiology at http://www.epidem.com/). Information on the STROBE Initiative is available at www.strobe-statement.org.

**Supplemental material 2.** Flow diagram of participant inclusion and data processing steps.

**Notes:** This figure illustrates the procedures of data cleaning and participant selection. It includes the merging and cleaning steps of three original datasets (OHIP questionnaire, clinical records, and dental examination data), followed by the exclusion of test records, missing values, participants without informed consent, and those under 18 years of age. Ultimately, 1,307 eligible participants were included in the study.
**Abbreviations:** OHIP: Oral Health Impact Profile; n: sample size.

**Supplemental material 3**. Baseline Characteristics by Periodontal Diagnosis

|  | *Healthy* | *Gingivitis* | *Periodontitis* | *p-value* |
| --- | --- | --- | --- | --- |
| *n* | 81 | 118 | 1054 |  |
| *Age (years) (mean (SD))* | 40.20 (14.79) | 45.05 (16.95) | 53.24 (13.32) | <0.001 |
| *Gender = Female* | 50 ( 61.73) | 76 ( 64.41) | 589 ( 55.88) | 0.112 |
| *Ethnicity* |  |  |  | 0.001 |
| *Caucasian* | 47 ( 58.02) | 86 ( 72.88) | 568 ( 53.89) |  |
| *Afro-Caribbean* | 12 ( 14.81) | 15 ( 12.71) | 242 ( 22.96) |  |
| *Asian* | 13 ( 16.05) | 15 ( 12.71) | 159 ( 15.09) |  |
| *Other-Mixed* | 5 ( 6.17) | 2 ( 1.69) | 63 ( 5.98) |  |
| *Smoking status* |  |  |  | <0.001 |
| *Never* | 58 ( 71.60) | 82 ( 69.49) | 532 ( 50.47) |  |
| *Former* | 14 ( 17.28) | 26 ( 22.03) | 330 ( 31.31) |  |
| *Current* | 8 ( 9.88) | 10 ( 8.47) | 187 ( 17.74) |  |
| *BMI (kg/m²) (mean (SD))* | 24.03 (4.64) | 25.71 (6.67) | 27.14 (7.00) | <0.001 |
| *Any systemic condition = Yes* | 32 ( 39.51) | 66 ( 55.93) | 588 ( 55.79) | <0.001 |
| *Diabetes = Yes* | 4 ( 4.94) | 10 ( 8.47) | 120 ( 11.39) | <0.001 |
| *Cardiovascular disease = Yes* | 6 ( 7.41) | 12 ( 10.17) | 125 ( 11.86) | <0.001 |
| *Hypertension = Yes* | 6 ( 7.41) | 22 ( 18.64) | 235 ( 22.30) | <0.001 |
| *Rheumatoid arthritis = Yes* | 2 ( 2.47) | 5 ( 4.24) | 68 ( 6.45) | <0.001 |
| *Number of teeth (mean (SD))* | 27.38 (5.23) | 26.84 (4.55) | 26.15 (4.87) | <0.001 |
| *Periodontal stage* |  |  |  | <0.001 |
| *Stage 0 (Healthy)* | 81 (100.00) | 118 (100.00) | 0 ( 0.00) |  |
| *Stage I* | 0 ( 0.00) | 0 ( 0.00) | 25 ( 2.37) |  |
| *Stage II* | 0 ( 0.00) | 0 ( 0.00) | 92 ( 8.73) |  |
| *Stage III* | 0 ( 0.00) | 0 ( 0.00) | 561 ( 53.23) |  |
| *Stage IV* | 0 ( 0.00) | 0 ( 0.00) | 353 ( 33.49) |  |
| *OHIP total score (mean (SD))* | 11.95 (11.88) | 12.87 (9.92) | 14.06 (11.43) | 0.319 |
| *OHIP-14: Occasionally to Very Often (Score≥2) (mean (SD))* | 3.64 (3.99) | 4.30 (3.57) | 4.63 (3.90) | 0.157 |
| *OHIP-14: Fairly Often or Very Often (Score≥3) (mean (SD))* | 1.40 (2.71) | 1.27 (2.06) | 1.67 (2.64) | 0.076 |
| *PISA score (mm²) (mean (SD))* | 54.96 (178.45) | 177.17 (417.45) | 495.18 (687.40) | <0.001 |

1.1.1.1. Note. Values are presented as number (percentage) unless stated otherwise, based on available (non-missing) data. Group comparisons were performed using one-way ANOVA or Kruskal–Wallis tests for continuous variables and chi-square tests or Fisher’s Exact tests for categorical variables, as appropriate. There were 58 (4.3%) participants with no information of the periodontal diagnosis hence omitted in the table. Other missing data included: BMI (7.3%), periodontal stage (5.9%), number of teeth (3.3%), ethnicity (2.0%), systemic and related conditions (≤1.1%), PISA score (0.8%), smoking (0.6%), and gender (0.3%). Abbreviations: BMI, body mass index; PISA, periodontal inflamed surface area; OHIP, Oral Health Impact Profile; OFOVO: Items rated "Occasionally", "Fairly Often", or "Very Often" (score ≥ 2); FOVO: Items rated "Fairly Often" or "Very Often" (score ≥ 3); RA, rheumatoid arthritis; CVD, cardiovascular disease; PD, periodontal disease.

1.1.1.2. Supplemental material 4. Correlation between PISA and OHIP Total Score in a) Periodontitis Group, b) Gingivitis group, c) Healthy group and d) stratified by diagnosis groups.

1.1.1.3. Notes: This scatter plot shows the relationship between PISA scores and OHIP-14 total scores in patients with periodontitis. Each dot represents an individual, and the red line indicates the fitted linear regression trend, with a shaded 95% confidence interval.
Abbreviations: OHIP-14: Oral Health Impact Profile-14; PISA: Periodontal Inflamed Surface Area; OHRQoL: Oral Health–Related Quality of Life.

1.1.1.4. Supplemental material 5. Correlation between BOP% and OHIP Total Score in a) Periodontitis Group, b) Gingivitis group, c) Healthy group and d) stratified by diagnosis groups.

1.1.1.5. Notes: This scatter plot displays the relationship between Bleeding on Probing percentage (BOP%) and OHIP-14 total scores among participants diagnosed with gingivitis. A linear regression line with a 95% confidence interval is overlaid to illustrate the trend.
Abbreviations: BOP: Bleeding on Probing; OHIP: Oral Health Impact Profile.

1.1.1.7. Supplemental material 6. Correlation between PISA and OHIP Total and Domains.

| OHIP domain | Overall (including healthy group) | Periodontitis Group | Gingivitis group | Healthy group |
| --- | --- | --- | --- | --- |
| 1) OHIP score | 0.06* | 0.06* | -0.08 | -0.07 |
| 2) Functional limitation | 0.04 | 0.04 | -0.12 | -0.02 |
| 3) Physical pain | 0.12*** | 0.13*** | -0.17 | -0.02 |
| 4) Psychological discomfort | 0.06* | 0.06 | -0.07 | -0.08 |
| 5) Physical disability | 0.03 | 0.03 | -0.18* | -0.07 |
| 6) Psychological disability | 0.05 | 0.06 | -0.05 | -0.07 |
| 7) Social disability | 0.04 | 0.04 | -0.02 | -0.07 |
| 8) Handicap | 0.03 | 0.02 | 0.02 | -0.09 |

Notes: This bar plot illustrates the correlation coefficients (ρ) between PISA scores and OHIP-14 total and domain-specific scores among participants with periodontitis. Among all domains, only Physical Pain showed a statistically significant positive correlation (ρ = 0.12, p < 0.001). Other domains and the total score exhibited weak or negligible associations with PISA, indicating limited monotonic relationships between inflammatory burden and perceived oral health-related quality of life in this group. Statistically significant estimates are marked with asterisks (*p < 0.05; **p < 0.01; ***p < 0.001).
Abbreviations: PISA: Periodontal Inflamed Surface Area; OHIP: Oral Health Impact Profile.

1.1.1.8. Supplemental material 7. Effect of Periodontal Diagnosis on OHIP Outcomes (Unadjusted and Adjusted Models)

1.1.1.9. Notes: This figure presents the estimated effect sizes (with 95% confidence intervals) of periodontal diagnosis (gingivitis or periodontitis, reference: healthy) on OHIP total and domain-specific scores. Results from both unadjusted (left panel) and adjusted (right panel) models are displayed. Adjusted models control for key covariates such as age, sex, ethnicity, and possibly other relevant confounders. The analysis shows that periodontitis has a positive effect on several OHIP scores, especially physical pain and total score. Statistically significant effects are indicated with asterisks (*p < 0.05). Gingivitis effects were generally smaller and not statistically significant.
Abbreviations: OHIP: Oral Health Impact Profile; CI: Confidence Interval.

**Supplemental material 8**. Effect of Periodontal Stage on OHIP Outcomes (Unadjusted and Adjusted Models)

**Notes**: This figure displays the estimated effect sizes and corresponding 95% confidence intervals for the association between periodontal stage (Stages I–IV, reference: healthy) and OHIP total and domain-specific scores. The left panel shows unadjusted models, while the right panel shows models adjusted for relevant covariates (e.g., age, sex, ethnicity). Results indicate a dose-response pattern, with Stage IV showing the strongest associations with higher OHIP scores, particularly in the domains of physical pain, psychological discomfort, and physical disability. Statistically significant estimates are marked with asterisks (*p < 0.05; **p < 0.01; ***p < 0.001).
**Abbreviations**: OHIP: Oral Health Impact Profile; CI: Confidence Interval.
